# Supplementary material for: The interactome of intact mitochondria by cross-linking mass spectrometry provides evidence for coexisting respiratory supercomplexes
Source: Mol Cell Proteomics. 2017 Dec 8;17(2):216–32. doi: 10.1074/mcp.RA117.000470 (PMC5795388; doi:10.1074/mcp.RA117.000470)
Supplement: Supplemental Data [file supp_RA117.000470_133922_0_supp_23978_fzffwf.zip › spectra_annotation/mito_spectra_annotation/51-1-1-1-17-1.pdf]

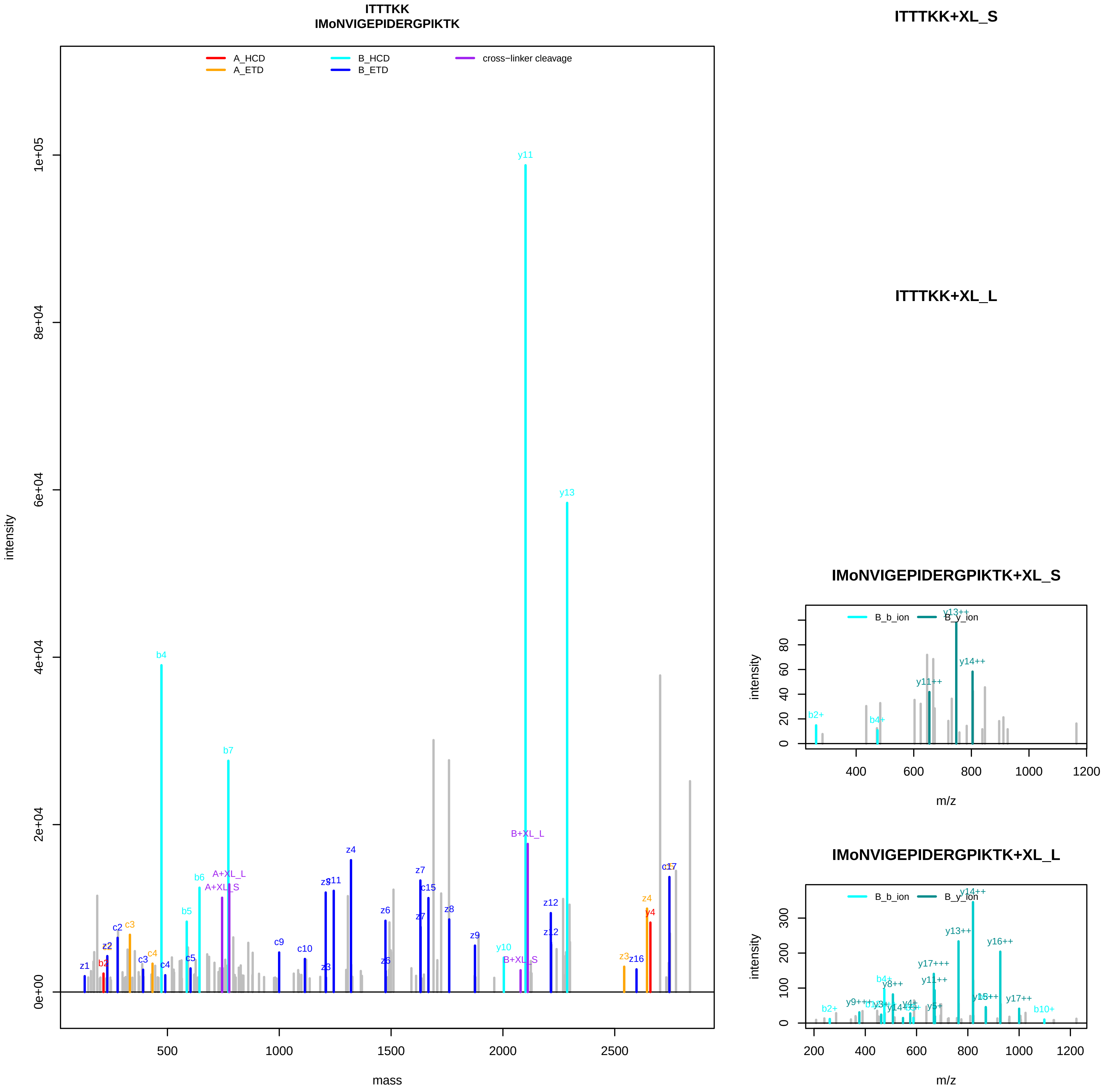
ITTTKK
IMoNVIGEPIDERGPIKTK
A_HCD
A_ETD
B_HCD
B_ETD
cross−linker cleavage
intensity
mass
0e+00
2e+04
4e+04
6e+04
8e+04
1e+05
500
1000
1500
2000
2500
y11
y13
b4
b7
b6
b5
A+XL_L
A+XL_S
B+XL_L
B+XL_S
z4
z7
c15
z8
z9
z12
c17
z4
y4
z3
z16
y10
c9
c10
c11
z6
c2
c3
c4
c5
z1
b2
z2
ITTTKK+XL_S
ITTTKK+XL_L
IMoNVIGEPIDERGPIKTK+XL_S
B_b_ion
B_y_ion
intensity
m/z
0
20
40
60
80
400
600
800
1000
1200
y13++
y14++
y11++
b2+
b4+
IMoNVIGEPIDERGPIKTK+XL_L
B_b_ion
B_y_ion
intensity
m/z
0
100
200
300
200
400
600
800
1000
1200
y14++
y13++
y16++
y17+++
y11++
b4+
y8++
y9+++
b2+
y17++
b10+
y15++
y5+
